# Inherited and somatic components in the pathogenetics of common diseases

**DOI:** 10.18699/vjgb-26-80

**Published:** 2026-09

**Authors:** M.S. Nazarenko, A.A. Sleptcov, V.P. Puzyrev

**Affiliations:** Research Institute of Medical Genetics, Tomsk National Research Medical Center of the Russian Academy of Sciences, Tomsk, Russia Tyumen Cardiology Research Center – Branch of the Tomsk National Research Medical Center of the Russian Academy of Sciences, Tyumen, Russia; Research Institute of Medical Genetics, Tomsk National Research Medical Center of the Russian Academy of Sciences, Tomsk, Russia Tyumen Cardiology Research Center – Branch of the Tomsk National Research Medical Center of the Russian Academy of Sciences, Tyumen, Russia; Research Institute of Medical Genetics, Tomsk National Research Medical Center of the Russian Academy of Sciences, Tomsk, Russia

**Keywords:** pathogenetics, common diseases, omnigenic model, atherosclerosis, cancer, germinal variants, somatic mutations, патогенетика, многофакторные заболевания, омнигенная модель, атеросклероз, злокачественные новообразования, герминальные варианты, соматические мутации

## Abstract

It is known that the pathogenesis of common diseases (CDs) is determined by a complex and nonlinear interaction of genetic predisposition, epigenetic modifications, and environmental factors. However, in addition to inherited genetic variants, somatic mutations play a key role in the pathological phenotype, shaping cell-type-specific genetic landscapes and influencing regional predisposition of target organs, age of onset, and variability in clinical manifestations. Thus, according to current concepts, CDs develop as a result of the combined influence of inherited germline variants and somatic mutations acquired during life, the interaction of which is realized through cell-specific molecular networks. Therefore, this review sequentially examines the main factors, classical and modern models of complex pathological phenotypes, as well as the achievements and prospects of analyzing inherited genetic variants in relation to CDs. The role of somatic mutations in the development of pathology is discussed using the example of atherosclerosis ontogenesis and the concept of athero-oncology through the assessment of somatic genomic variability in smooth muscle cells of atherosclerotic plaques, where the sequential accumulation of “driver” mutations confers a selective advantage and triggers clonal evolution in the tissue microenvironment. Special attention is given to the concept of “paradominant” inheritance – a special form of non-Mendelian transmission of complex traits that combines inherited predisposition and somatic mutations that arise early in ontogenesis. Based on an analysis of current data, we propose an integrative hypothesis postulating that inherited genetic variants form a body-wide predisposition to CDs, while somatic mutations, arising and selectively increasing in specific cellular compartments of target organs, are critical events explaining the regional specificity, temporal dynamics (age of onset), and variability in the severity of the clinical phenotype. A new paradigm for predictive medicine for CDs is based on a comprehensive characterization of the continuum of genetic variants (inherited and somatic), an analysis of their interactions with cell-specific molecular networks, and a transition from population-based risk assessments to causal models of individual pathogenesis.

## Abstract (ru)

Хорошо известно, что патогенез многофакторных заболеваний определяется сложным и нелинейным взаимодействием генетической предрасположенности, эпигенетических модификаций и факторов внешней среды. Однако помимо унаследованных генетических вариантов, важную роль в развитии патологического фенотипа играют соматические мутации, образуя клеточно-специфичные генетические ландшафты и влияя на регионарную предрасположенность органов‑мишеней, возраст манифестации и вариабельность клинических проявлений. Таким образом, согласно современным представлениям, многофакторные заболевания формируются в результате совместного влияния унаследованных герминативных вариантов и соматических мутаций, приобретенных в течение жизни, взаимодействие которых реализуется через клеточно-специфичные молекулярные сети. В связи с этим в обзоре последовательно рассматриваются основные факторы, классические и современные модели сложных патологических фенотипов, а также достижения и перспективы анализа наследуемых генетических вариантов в отношении многофакторных заболеваний. Роль соматических мутаций в формировании патологии обсуждается на примере онтогенеза атеросклероза и концепции атеро-онкологии через оценку соматической вариабельности генома в гладкомышечных клетках атеросклеротической бляшки, когда последовательное накопление «драйверных» мутаций дает селективное преимущество и запускает процесс клональной эволюции в тканевом микроокружении. Отдельное внимание уделено описанию концепции «парадоминантного» наследования – особой форме неменделевской передачи сложных признаков, сочетающей унаследованную предрасположенность и соматические мутации, возникающие на ранних стадиях онтогенеза. На основе анализа современных данных нами предлагается интегративная гипотеза, постулирующая, что унаследованные генетические варианты формируют общеорганизменный фон предрасположенности к многофакторным заболеваниям, в то время как соматические мутации, возникающие и селективно увеличивающиеся в специфических клеточных компартментах органов- мишеней, являются критичным событием, объясняющим топическую специфичность, временнýю динамику (возраст манифестации) и вариабельность тяжести клинического фенотипа. Новая парадигма предиктивной медицины в отношении многофакторных заболеваний основывается на комплексной характеристике континуума генетических вариантов (унаследованных и соматических), анализе их взаимодействия с клеточно‑специфичными молекулярными сетями и переходе от популяционных оценок риска к каузальным моделям индивидуального патогенеза.

## Introduction

Pathogenetics is a discipline that studies the etiological role of hereditary factors and the molecular genetic mechanisms underlying the initiation, development and progression of pathological processes. It focuses on the causal pathways through which a genetic predisposition is transformed into a clinical disease phenotype (Puzyrev, Kucher, 2011). This transformation is considered through the prism of the interaction of genetic variants with epigenetic regulatory systems and networks of intergenic interactions. Moreover, pathogenetics includes the description of processes occurring at all levels of biological organization, from the molecular to the population level, within the framework of interaction with the environment.

The fundamental goal of pathogenetics in medicine is to establish causal relationships between genetic variants and pathological phenotypes, thereby providing the basis for the development of pathogenetically grounded diagnostic strategies, targeted therapies, and preventive interventions (Puzyrev, Kucher, 2011). The greatest progress in this field has been achieved in the study of monogenic and chromosomal diseases. However, the pathogenetics of common diseases (CDs) remains, to a large extent, an insufficiently studied area.

Since the beginning of the era of genome-wide studies, the formal genetic approach to the investigation of CDs, which was primarily based on heritability estimates derived from twin and family studies, has been complemented by the paradigm of medical genomics. This new paradigm was aimed at identifying specific genetic loci and structural variants associated with the predisposition to CDs from evolutionary and ontogenetic perspectives (Puzyrev, Kucher, 2011).

The subsequent methodological shift, which marked the transition from the cataloging of genetic associations to the construction of causal models, required the convergence of genome-wide sequencing, functional genomics, and systems biology data. This integration enabled the interpretation of identified genetic associations through the prism of cell typespecific regulatory networks, epigenetic mechanisms, and a hierarchy of pathophysiological pathways that determine disease trajectories.

The convergence of these methodological approaches has shaped the modern paradigm of CD pathogenetics, in which critical importance is attributed not only to the spectrum of genetic variants but also to their ontogenetic origin. In this framework, genetic variants are classified as either inherited germline variants or de novo somatic mutations, each making fundamentally distinct contributions to disease penetrance, age at onset, and clinical polymorphism.

In this regard, the aim of this review is to examine the inherited and somatic components involved in the pathogenetics of CDs.

## Common diseases: factors and models

According to current concepts, CDs are the result of complex nonlinear interactions in the “genome–epigenome–environment” system. The intensity with which each of these components has been investigated varies for historical reasons and is largely determined by the level of development of biomolecular and bioinformatic technologies. Consequently, the dominant research paradigms in this field have consistently shifted from classical genetics toward integrative analyses of polygenic networks and their interactions with environmental factors.

Approaches to identifying predisposition loci for CDs have evolved from paradigms based on candidate gene analysis and microarray-based genome-wide association studies (GWAS) to population-specific analysis of whole genome sequencing data with the detection of the full spectrum of genetic variation, from common to unique variants, in combination with in vivo and in vitro functional genomics approaches. These paradigms are further complemented by epigenomic annotation maps for prediction of cell typespecific regulatory effects and identification of causal genes.

An important aspect of understanding genotype-phenotype relationships in CDs is determining how genetic variants influence gene regulation and expression across different tissues and cell types. Such analyses may be performed using in silico bioinformatic approaches or through the integration of GWAS data with epigenomic and transcriptomic datasets, including single-cell datasets.

The central concept in the study of CDs is genetic predisposition, defined as an integral probabilistic measure that quantifies the contribution of polygenic burden, inherited rare high-penetrance variants, and the modifying effects of environmental factors to the individual profile of the disease risk. This profile is described using probability distribution functions for different clinical phenotypes while accounting for the temporal dynamics of phenotype manifestation.

According to established models, the formation of predisposition to CDs is described within the framework of either polygenic or oligogenic architectures. The polygenic architecture involves the additive effects of numerous genetic variants (polygenes), each exerting a small effect, whereas the oligogenic architecture is characterized by a limited number of driver genes with substantial effects on penetrance. In both cases, additional genetic and epigenetic modifiers influence disease onset and the severity of the clinical phenotype (Fig. 1).

**Fig. 1.**
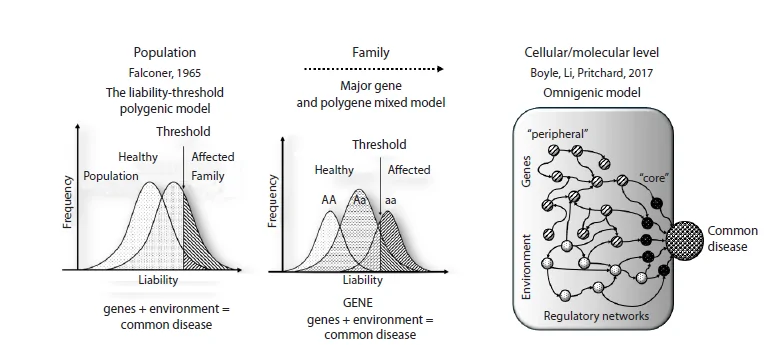
Evolution of inheritance models for common diseases.

A key implication of the liability-threshold polygenic model is that genetic predisposition alone does not inevitably result in disease development but only shifts the distribution of disease risk within a population. Meanwhile, clinical manifestation occurs when the individual multifactorial burden exceeds a certain systemic threshold of tolerance, determined by the reserve capacity of compensatory physiological mechanisms.

One of the influential hypotheses proposed to explain the complex genetic architecture of CDs is the omnigenic model (Boyle et al., 2017) (Fig. 1). According to this concept, a large repertoire of genetic variants, predominantly exerting cis-regulatory effects, modulates the expression of genes functioning on the periphery of pathogenetic pathways. These dysregulated transcription programs are integrated through complex trans-regulatory networks, converging in the altered activity of a limited number of highly conserved core genes that directly determine key pathophysiological processes. It should be emphasized that the dichotomy of “core” and “peripheral” genes itself is functional and contextdependent rather than strictly structural, since the same gene may function as a “core” gene in one tissue-specific network and as a “peripheral” gene in another. Statistically significant GWAS associations are detected predominantly for “peripheral” genes because such variants have higher population frequencies and because current statistical approaches possess limited statistical power to detect the effects of rare variants located within “core” gene loci.

The omnigenic model provides a unified explanation for the key features of the genetic architecture of CDs, including high polygenicity, the predominance of low-effect variants, widespread pleiotropy, and the critical importance of regulatory interactions mediated by transcriptional networks. This model claims to be a conceptual shift in the interpretation of the genetic architecture of CDs, offering a mechanistic explanation of polygenicity through the prism of regulatory convergence. However, its full verification requires a transition from correlational expression maps to direct experimental demonstration of causal relationships within regulatory networks, for example through Mendelian randomization approaches or gene-editing technologies, as well as taking into account the contribution of non-transcriptional mechanisms such as post-translational modifications and metabolic control.

Despite its heuristic value, the omnigenic model remains a simplified representation and leaves several fundamental questions to be solved. What objective biomolecular and functional criteria should be used to stratify genes into “core” and “peripheral” categories and to identify them reliably across different tissues and ontogenetic stages? Are pathogenetically relevant regulatory networks exclusively intracellular, or do they fundamentally incorporate intercellular signaling cascades that form the tissue-specific disease contexts? How can the contribution of converging regulatory influences be quantified relative to direct causal effects in specific cell types? Furthermore, in its current formulation, the model does not fully account for the role of chromatin spatial organization and intercellular communications in the formation of an integral pathogenetic context.

## Inheritable genome and common diseases

Modern studies of the genetic architecture of CDs, initially based on GWAS, have cumulatively identified a vast repertoire of statistically significant associations between genetic variants, primarily single nucleotide polymorphisms (SNPs), and diseases catalogued in repositories such as NHGRI-EBI GWAS Catalog (https://www.ebi.ac.uk/gwas/). Analysis of these data has revealed the key features of such associations.

The vast majority of identified genetic associations are characterized by small effect sizes and increase the odds ratio (OR) of disease development to approximately 1.1–1.5, corresponding to a relative risk increase of 10–50 %. As a rule, a single common genetic variant (minor allele frequency [MAF] > 1–5 %) explains only a small proportion of heritability for most CDs, which limits its clinical utility as an isolated prognostic biomarker.

Cohort and prospective studies are currently being conducted to refine these risk estimates and to elaborate methods for genetic risk stratification. To maximize the statistical power of GWAS and minimize systematic errors resulting from population stratification, it is critically important to form large, phenotypically and genotypically well-characterized cohorts that adequately reflect the ethnic background of study participants. Such cohorts are also essential for the elaboration and calibration of polygenic risk scores (PRSs) relevant to specific populations, since differences in allele frequencies and haplotype structure between populations substantially limit the applicability of scores created on a different genetic background.

In the Russian Federation, the elaboration of populationspecific PRS is a topic of active research, with research groups led by E.K. Khusnutdinova (Academician of the Bashkortostan Academy of Sciences), O.M. Drapkina (Academician of the Russian Academy of Sciences, RAS), M.I. Voevoda (Academician of RAS), A.V. Polonikov (Doctor of Research Medicine), A.N. Meshkov (Doctor of Research Medicine), M.I. Churnosov (Doctor of Research Medicine), V.E. Golimbet (Doctor of Biological Sciences), A.O. Kibitov (Doctor of Research Medicine), and researchers from the Centre for Strategic Planning of the Federal Medical and Biological Agency of Russia.

It has been established that combinations (ensembles) of allelic variants of various genes may substantially increase both disease susceptibility and the probability of disease complications. The combined effect of such polygenic combinations, aggregated into PRSs, may explain a considerable proportion of heritability for certain CDs; however, their contribution varies significantly among diseases and depends on the ethnic composition of the studied population. Nevertheless, a substantial fraction of CD heritability remains unexplained within the framework of the “common disease-common variant” model, a phenomenon referred to as “missing heritability”. Current concepts suggest that this missing component reflects the combined contribution of rare genetic variants, structural genomic rearrangements, epistatic interactions. The limitations of statistical models used to estimate heritability also play a role, as they do not fully account for the contribution of somatic mutagenesis and inaccuracies in phenotyping.

Whole genome sequencing (WGS) and whole exome sequencing (WES) are critical for identifying a range of rare and unique variants, including single nucleotide variants (SNVs) and copy number variations (CNVs). To evaluate their contribution to the “missing heritability” and establish associations with disease phenotypes, specialized statistical approaches are used, such as the transmission disequilibrium test (TDT) for de novo mutation analysis and variant aggregation methods (burden test, SKAT), which assess the cumulative effects of rare alleles within genes or biological pathways.

The variants associated with CDs are located in the nuclear and mitochondrial genomes, highlighting the complexity of the functional interactions underlying the pathogenesis of the respective diseases. Genetic studies have revealed widespread pleiotropy, in which individual genetic loci and specific variants are associated with the risk of developing several CDs, which can vary in their clinical manifestations. This phenomenon provides a molecular basis for genetic correlations observed clinically among diseases, and for phenomena such as syntropy (comorbidity) (Bragina, Puzyrev, 2023). These findings indicate the presence of shared molecular mechanisms in the pathogenesis of various CDs and open new perspectives for understanding their genetic architecture.

The GWAS results demonstrate that the vast majority of significant SNPs are located within non-coding genomic regions enriched with epigenetic marks of active regulatory elements such as enhancers and promoters. This observation suggests that SNPs predominantly influence CD risk through indirect regulatory mechanisms, including allele-specific effects on transcription factor binding and subsequent dysregulation of target gene expression, rather than through direct alterations in protein structure. However, the interpretation of functional significance of SNPs requires caution because of potential linkage disequilibrium with truly causal variants, including rare or structural variants.

Moreover, despite the large number of identified associations, the “causal” or “driver” genetic variants in relation to CDs often remain unknown. Consequently, modern studies of the CD pathogenesis rely on integrative systems biology approaches aimed at constructing causal models through the combined analysis of multi-omics datasets. However, the key methodological challenge remains not simply the integration of heterogeneous datasets (genome, epigenome, translatome, transcriptome, proteome, metabolome, and exposome), but also the reconstruction of the temporal sequence and hierarchical organization of molecular events within pathogenetic cascades, as well as taking into account the spatial organization of the genome and intracellular compartments that determine the specificity of molecular interactions.

Furthermore, the relationship between genetic variants and CDs is now being analyzed not only for individual diseases but also for patterns of disease co-occurrence (syntropy) and mutual exclusion (dystropy), as well as within the broader framework of the diseasome, which conceptualizes diseases according to their shared molecular networks and genetic relatedness (Bragina, Puzyrev, 2023). However, accurate quantification of the diseasome requires more complex metrics than the simple enumeration of shared loci.

Generalizations within the framework of the study of the phenomenon of disease combinations provide an opportunity to approach the systematization and natural classification of diseases based not on organ systems or clinical symptoms, but rather on the commonality of their underlying molecular networks and pathogenetic pathways. This approach opens up prospects for the development of fundamentally new therapeutic strategies targeting key nodes shared among multiple diseases rather than individual nosological entities.

The strategy of deconvolution of the molecular genetic mechanisms of CDs requires integrative profiling of a broad spectrum of genetic variants (SNV, CNV, mobile elements), epigenetic landscapes (DNA methylation, histone modifications, chromatin accessibility patterns) and gene expression profiles obtained with single-cell resolution and spatialtemporal organization in target organ tissues using single-cell multi-omics and spatial transcriptomics technologies.

The statistical analysis of such sparse data and identification of biologically meaningful patterns remains methodologically challenging. A key step is the integration of GWAS data with multi-omics profiles of relevant cell types to infer causal genes and cell-specific regulatory pathways. However, the success of such approaches fundamentally depends on the completeness and accuracy of cellular atlases and annotations of functional genomic elements for specific human tissues, since incomplete or inaccurate reference datasets may lead to distorted conclusions regarding causality.

Historically, most studies of CD genetics have focused on inherited germline variants, primarily SNPs, analyzed in easily accessible surrogate tissues such as peripheral blood. This approach imposes substantial limitations because it underestimates the tissue and cell specificity of molecular processes, as well as the dynamics of transcriptional and epigenetic states during ontogenesis. Such studies are based on the assumption that most cells in the human body have an identical genome and are genetically stable throughout ontogenesis. However, this traditional perspective ignores the growing body of evidence indicating a significant contribution of somatic mutations to CD pathogenesis, which manifests through the formation of mosaic genetic landscapes in target tissues (dysfunction of tissue-specific cell pools) and can significantly modify the phenotype determined by germinal variants. The role of somatic mutations in the development of CDs may be illustrated using the example of atherosclerosis.

## Somatic genome variability in atherosclerotic plaque smooth muscle cells

The pathogenesis of atherosclerosis may be viewed through the prism of clonal expansion of somatic cells carrying pathogenic genetic variants within the arterial wall. In this context, atherogenesis demonstrates conceptual parallels with the model of carcinogenesis, in which the sequential accumulation of “driver” mutations gives a selective advantage and promotes clonal evolution within the tissue microenvironment. It is well known that atherosclerosis and malignancy share numerous common features, from a prolonged latent period and overlapping risk factors to similar molecular and cellular processes and mechanisms (Baylis et al., 2023).

At the molecular level, these similarities are reflected in the common characteristics of the microenvironment, including chronic unresolved inflammation, hypoxia, angiogenesis and extracellular matrix remodeling. They also involve fundamental cellular phenotypes such as the metabolic shift towards aerobic glycolysis, resistance to apoptotic signals, dysregulation of cellular senescence mechanisms, escaping immune surveillance in a manner similar to cancer stem cell (CD47 expression, impairment in efferocytosis), epithelialmesenchymal transition, increased invasiveness and, most importantly, clonal expansion driven by somatic mutations (DiRenzo et al., 2017).

The discovery of monoclonality of smooth muscle cells (SMCs) within atherosclerotic plaques formed the basis for the hypothesis that atherogenesis represents a neoplastic process in which a series of somatic driver mutations, for example in cell cycle regulatory genes (TP53, NOTCH1), determines the clonal expansion of cells with a selective advantage (Benditt E., Benditt J., 1973). According to this hypothesis, the initiation and progression of atherosclerotic lesions are associated with the accumulation of somatic mutations induced by both endogenous processes (oxidative stress, replication errors) and exogenous factors (chemical mutagens, oncogenic viruses), which creates the molecular basis for clonal evolution within the vessel wall. Although the monoclonal theory of atherogenesis was proposed a long time ago, experimental studies aimed at its verification began to emerge only after 2015.

The validation of the monoclonal hypothesis of atherosclerosis became possible through the development of high-resolution approaches, such as lineage tracing in animal models, as well as single-cell multi-omics analysis (scRNA-seq, scATAC-seq) combined with spatial transcriptomics technologies. These approaches make it possible not only to visualize the clonal architecture of the plaque, but also to establish causal relationships among specific somatic mutations, clonal dynamics, and pathogenic transcriptomic programs, thereby revealing the sequence of cellular and molecular events underlying disease development.

Application of an integrated methodological approach has demonstrated that phenotypically altered SMCs within atherosclerotic plaques accumulate oncogenic “driver” mutations and exhibit properties resembling those of tumor cells, including disturbances in cell cycle regulation and apoptosis mechanisms. These findings provide direct experimental support for the hypothesis of a deep commonality of the fundamental molecular and cellular mechanisms underlying atherogenesis and carcinogenesis.

SMCs and their derivatives within atherosclerotic plaques exhibit accumulation of oxidative DNA damage, which serves as a source of somatic mutations and leads to an increase in genomic instability that correlates with the extent of SMC transdifferentiation toward macrophage-like and mesenchymal cells. Phenotypically altered SMCs also harbor somatic copy number variants (CNVs) affecting oncogenes (EGFR, KRAS) and tumor suppressor genes (FAT1, UBR5), as well as genes associated with coronary artery disease (CCM2, PALLD). Notably, the spectrum of these genomic alterations overlaps with that observed in solid tumors. The oncogenic KrasG12D mutation accelerates SMC phenotypic switching and promotes atherosclerosis progression (Pan et al., 2024).

According to the current paradigm, mature SMCs undergo dedifferentiation in response to pathogenic stimuli and give rise to oligoclonal populations. These clones migrate from media to intima and form a fibrous plaque cap, but their subsequent fate is heterogeneous. Some clones transdifferentiate into macrophage-like phenotypes that promote inflammation and destabilization, whereas others acquire osteogenic properties leading to vascular calcification. Together, these processes influence plaque structural integrity and susceptibility to rupture. Thus, clonal expansion of SMCs represents a dynamic pathological process that may initially contribute to formation of a stabilizing fibrous cap during the early stages of atherogenesis, but later promotes plaque destabilization through the accumulation of additional somatic mutations and activation of pro-inflammatory transdifferentiation programs, thereby increasing the risk of plaque rupture and acute vascular events.

The molecular basis of SMC clonal expansion may involve the existence of discrete subpopulations of progenitor cells within the vascular wall characterized by unique transcriptomic and epigenetic landscapes that determine enhanced proliferative capacity and resistance to apoptosis under conditions of atherogenic stress. In particular, experimental studies have demonstrated that the C3high Sca1+ SMC subpopulation functions as a key regulatory node. These cells not only promote autonomous SMC proliferation through autocrine signaling but also mediate macrophage recruitment and functional reprogramming toward a pro-inflammatory phenotype characterized by impaired efferocytosis, thereby enhancing the inflammatory response and exacerbating tissue damage (Dobnikar et al., 2018).

An alternative model suggests that, although all SMCs of media initially possess proliferative potential, clonal dynamics is determined by the selection of subpopulations that acquire a selective advantage through somatic mutations, enabling hyperproliferation and evasion of efferocytosis. In parallel, selective advantage may also result from increased tolerance to a pathological environment characterized by metabolic stress and inflammation, or through mechanisms of lateral inhibition in which dominant clones actively suppress neighboring cells via short-range (juxtacrine) signaling (Luo et al., 2023).

Accumulating evidence suggests that the contribution of structural mutations themselves to SMC phenotypic alterations may be limited. Instead, epigenetic reprogramming, including alterations in DNA methylation and histone modifications, may function as an alternative or synergistic mechanism underlying SMC clonal expansion. In this case, SMC clonality may be partially explained by dynamic changes in the chromatin architecture that contribute to maladaptive cellular responses to inflammatory stress. Notably, the transcription factor ATF3 has been identified as a key suppressor of SMC transition toward a pro-inflammatory phenotype. Its protective effect is realized not through the activation of anti-inflammatory programs, but rather through the direct suppression of pro-inflammatory signaling pathways, particularly through the inhibition of the complement activation cascade (Wang et al., 2022).

The molecular mechanisms of SMC clonal expansion may be mediated by intercellular interactions in the macrophage– SMC axis. Moreover, myeloid cells carrying functional defects, such as decreased expression of the epigenetic regulator TET2 and integrin β3, generate a pro-inflammatory environment through hyperactivation of the TNF signaling pathway. This process contributes not to oligoclonal but to polyclonal proliferation of SMC progenitor cells and the formation of more extensive atherosclerotic lesions (Kabir et al., 2023).

The heterogeneity of SMC transcriptional profiles, determined by their embryonic origin (Dobnikar et al., 2018), may underlie the variable potential for clonal expansion among various segments of the arterial bed, which, in turn, may explain the regional predisposition to pathological lesion formation. It has been hypothesized that clonal expansion of vascular cells, including SMCs, endothelial cells, and macrophages, in various vasculopathies (atherosclerosis, aneurysm, pulmonary hypertension, and cavernous malformation of the brain) may be regulated by conserved molecular pathways shared across distinct diseases (Lin et al., 2023). These pathways are likely integrated with fundamental ontogenetic programs that determine cellular plasticity and proliferative potential of the cells.

Moreover, it has been demonstrated that the epigenomic landscape, particularly chromatin accessibility, in individual cells (SMCs, fibroblasts, endothelial cells) within different segments of the ascending aorta is determined by their embryonic origin. These ontogenetically inherited regulatory domains spatially co-localize with cardiovascular disease risk loci identified in genome-wide association studies (GWAS). In particular, studies have shown that in normal mice the transcriptomic and epigenomic landscapes of individual arterial cells (SMCs, fibroblasts, and endothelial cells) depend on cell type and vascular bed, and the “open chromatin” regions are enriched with human cardiovascular disease risk genes (Weldy et al., 2025).

It has been assumed that enhancers of genes of developmental transcription factors (Tbx20, Hand2, Gata4, and Hoxb), which are specific to particular cell types and vascular regions, serve as a key element in the interpretation of complex genetic risk factors for arterial pathology by transforming inherited polymorphisms into cell-specific pathological programs (Weldy et al., 2025). At the same time, aneurysm/ dissection and atherosclerosis of the ascending aorta in humans are associated with multidirectional alterations in DNA methylation patterns within regulatory regions linked to the transcription factor genes NR2F1 and TBX20, which play a key role in the vascular development in embryogenesis. These findings indicate reactivation of ontogenetic programs during disease pathogenesis (Koroleva et al., 2024).

Thus, the cumulative and dynamic interaction of multiple genetic and epigenetic factors affecting cellular and molecular systems throughout ontogenesis forms a pathological context that promotes disease comorbidity. Within this context, somatic variants, including both structural genomic alterations and persistent epigenetic reprogramming, function as universal mechanisms modifying cellular phenotypes and integrating pathogenic influences.

Somatic mutations are detected not only in parenchymal cells of target organs in CDs, but also in hematopoietic cells, where they manifest as clonal hematopoiesis (CH). Of particular clinical and biological significance is clonal hematopoiesis of indeterminate potential (CHIP), characterized by the presence of a “driver” mutation in the absence of diagnostic criteria for hematologic neoplasm (Jaiswal et al., 2014). Intensive study of CHIP has revealed its role as a systemic pro-inflammatory factor mediating the effect of somatic mosaicism on the pathogenesis of a broad range of CDs through the secretion of pro-inflammatory cytokines by mutant macrophage clones and other myeloid-derived cells (Schuermans, Honigberg, 2025).

However, the detailed molecular and cellular mechanisms linking CHIP to such a wide range of CDs remain incompletely understood. The concept of “paradominance”, in which a somatic mutation occurring on the background of an inherited heterozygous mutation in the same gene (for example, TET2, DNMT3A) leads to a complete loss of function and subsequent manifestation of a pathological phenotype, represents an important conceptual bridge between classical genetics and the genetics of somatic cells. This concept offers a mechanistic explanation for cases of incomplete penetrance and mosaic expressivity that cannot be adequately explained by traditional inheritance models and may be particularly relevant for genes in which constitutional homozygous inactivation is embryonically lethal.

## The concept of “paradominant” inheritance in relation to common diseases

“Paradominant” inheritance represents a distinct form of non-Mendelian inheritance that combines inherited predisposition with somatic mutations acquired during ontogenesis (Happle, 1991, 1993). Unlike classical Mendelian models, this mechanism assumes that the pathological phenotype manifests only when an inherited heterozygous germline mutation is followed by a somatic mutation resulting in loss of heterozygosity (LOH) of the remaining wild-type allele in progenitor cells. This process leads to clonal expansion of cells carrying biallelic gene inactivation. Importantly, constitutional homozygosity for such mutations is usually embryonically lethal (Fig. 2), which explains why these alleles persist in the population primarily in the heterozygous state and manifest phenotypically only after somatic second-hit events.

**Fig. 2.**
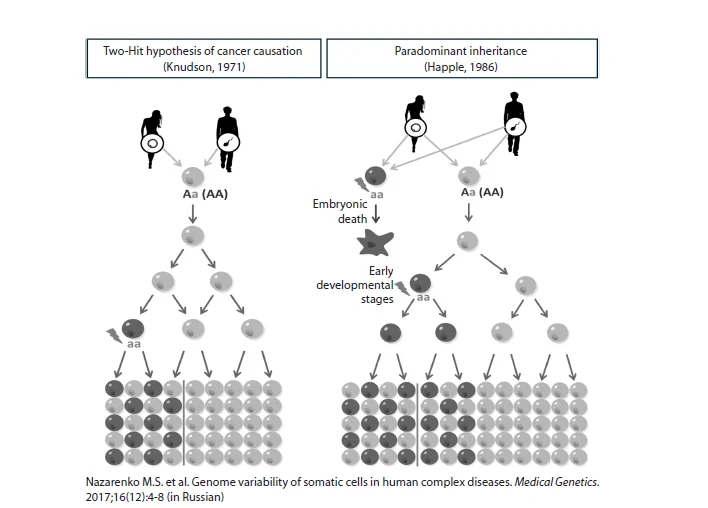
Molecular mechanisms of somatic mutations

A key feature of “paradominant” inheritance is the formation of a mosaic genotype resulting from LOH events occurring in a subset of somatic cells at an early stage of embryo development. Interestingly, similar mosaic alterations may arise independently in multiple members of the same family, thereby mimicking autosomal dominant inheritance despite a fundamentally different underlying mecha-nism. This phenomenon formed the basis of the term “paradominance”.

Unlike the Knudson model, according to which constitutional heterozygosity for non-lethal mutations predisposes individuals to tumor development, paradominance is characterized by constitutional heterozygosity specifically for embryonically lethal mutations, which excludes Mendelian inheritance and manifests exclusively through somatic mosaicism. In this context, the somatic event – most commonly post-replicative mitotic recombination – occurs at an early stage of embryogenesis. The specific time point of mutation occurrence determines both the size and tissue prevalence of the mutant clone, which in turn determine not only the mosaic pattern of abnormal cell distribution but also the extent of tissue involvement, which directly influences clinical severity (Happle, 2009).

Initially, this concept was proposed to explain the pathogenesis of several dermatological syndromes, such as McCune– Albright syndrome and Becker’s pigmented nevus (Happle, 1991, 1993). A classic example is provided by multifocal venous malformations, in which biallelic somatic mutations in the TEK (receptor tyrosine kinase) gene arise in the background of an inherited germline p.R849W mutation. This combination results in autonomous receptor activation and local clonal expansion of endothelial cells, highlighting the importance of targeted analysis of somatic variants directly in pathological tissues (Limaye et al., 2009). However, subsequent studies have revealed the role of “paradominant” inheritance in other pathologies, including atherosclerosis (Fig. 2).

Modern studies employing high-resolution sequencing technologies and mosaicism analyses are likely to clarify the contribution of “paradominant” mechanisms to the pathogenesis of CDs, particularly in diseases characterized by sporadic occurrence, familial clustering, and mosaic patterns of organ involvement that cannot be adequately explained by classical inheritance models.

## Translational potential in the practice of common diseases research

At the current stage of development, genetic testing for hereditary predisposition to CDs is characterized by its gradual integration into clinical practice. The elaboration of population-specific PRSs based on genome-wide data enables stratification of individuals in cohorts for predictive assessment of the risk of developing CDs, their complications, and potential response to pharmacotherapy. However, the widespread clinical implementation of these approaches remains limited by the low reproducibility of predictive power when used in genetically distinct populations.

When whole-exome sequencing (WES) or whole-genome sequencing (WGS) is performed in CD cohorts, analysis of rare genetic variants in individuals may facilitate the identification of novel molecular targets for pharmacological intervention. Moreover, it has been demonstrated that combining common and rare genetic variants within a single model improves the accuracy of CD risk prediction (Fiziev et al., 2023).

The integration of the so-called “non-genetic” determinants of CDs – including epigenetic markers, exposome parameters, as well as their complex interactions, analyzed within the framework of multi-omics approaches and comorbid pattern analysis, – into unified predictive models has considerable, although methodologically more complex, translational potential compared with purely genetic scales.

Understanding the prevalence, spectrum, and mechanisms underlying CHIP in CDs is important for the development of novel screening strategies, individual risk assessment and the implementation of diagnostic and treatment programs. For example, an individual prognosis may be established through integration of traditional risk factors and clinical status with the molecular characteristics of CHIP. The type of somatic mutation and VAF are of key importance. An individual with large TET2 CHIP (>10 % VAF) and borderline 10-year risk of cardiovascular complications would be counseled regarding aggressive risk reduction with intensification of lifestyle modifications and aggressive lipid-lowering and anti-hypertensive therapies. In contrast, such interventions are not indicated in individuals with DNMT3A CHIP and a low 10-year CVD risk. While there are currently no randomized clinical trials specifically supporting these interventions on the basis of CHIP status, therapeutic tactics relies on shared decision-making, when the doctor and the patient carefully balance potential benefits and risks (Oren, Libby, 2025). Translation of findings from fundamental studies investigating the role of somatic genetic variants in target organ cells in CDs into clinical practice is not so obvious.

Targeting the pathogenetic mechanisms underlying clonal expansion represents a promising but methodologically complex direction for the development of novel therapeutic strategies for CDs. Such approaches include the selective elimination of mutant hematopoietic stem cell clones, for example, through senolytic therapies, modulation of the pro-inflammatory phenotype of smooth muscle cell clones, or inhibition of specific inflammatory mediators produced by these clones.

The proof of the fundamental possibility of such targeted therapeutic approaches is the atheroprotective effect observed for some antitumor agents. These include the blockade of the “don’t eat me” signaling pathway using anti- CD47 antibodies to enhance the efferocytosis of apoptotic plaque cells (Kojima et al., 2016; Tao et al., 2020); inhibition of SMC clone proliferation using tretinoin (Pan et al., 2020); and the use of selective vulnerability of SMCs with impaired DNA repair through the use of PARP inhibitors such as niraparib (Pan et al., 2024). These findings open new prospects for the repositioning of oncological drugs in cardiovascular medicine.

## Conclusion

The evolution of CD pathogenetics demonstrates a shift from the identification of disease-associated loci to the systemic functional characterization of cellular phenotypes driven by these genetic variants within cell-specific molecular networks, which marks the transition from genetic association to a causal understanding of pathogenesis.

The pathogenesis of CDs is characterized by multifactorial nature and is realized through nonlinear dynamic interactions between genetic predisposition, epigenetic modifications and environmental factors, forming complex causal networks. In this regard, progress in the prediction, diagnosis, treatment and prevention of CDs in the context of personalized medicine requires not merely the accumulation of data, but the elaboration of fundamentally new computational and conceptual models capable of integrating heterogeneous causal factors and their nonlinear interactions at various levels of biological organization, from the molecular to the organismal level, in order to construct predictive dynamic models of individual pathogenesis.

Somatic mutations that form mosaic genetic landscapes (somatic mosaicism) at the level of intra- and interstitial heterogeneity, together with tissue- and cell-specific epigenetic programs, act as key modifiers of penetrance, expressivity, and clinical polymorphism in CDs. Since the burden and spectrum of somatic mutations, including “driver” events, accumulate in a tissue-specific manner throughout life during ontogenesis, this process represents a universal mechanism explaining such fundamental characteristics of CDs as the regional predisposition of target organs and variable expressivity in different individuals. At the same time, the spatial distribution and clinical manifestation of these mutations critically depend on local selective processes and the rate of cellular turnover within each specific tissue.

The greatest success in characterizing the frequency and spectrum of somatic mutations at the molecular level in target organ cells and tissues in “non-cancerous” CDs has been achieved for SMCs in atherosclerosis. The key tasks remain unresolved: the systematic identification and functional verification of the full repertoire of “driver” somatic mutations, the determination of the contribution of structural variants and mobile elements to pathogenesis, as well as the full-scale characterization of the frequency and spectrum of somatic mosaicism across different cell types (populations of macrophages, endotheliocytes, smooth muscle cells, neurons) in a broad range of CDs.

Genotype-specific patterns of clonal hematopoiesis (CHIP) determined by mutations in distinct “driver” genes (TET2, DNMT3A, JAK2, ASXL1) are associated with divergent cardiovascular risk profiles, indicating the need for the development of targeted therapeutic interventions. Studies in cellular and mouse models, in particular using bone marrow transplantation from mutant animals, confirm the causal relationship between CHIP and cardiovascular diseases. This relationship is realized through a complex of alterations, including a loss of balance between self-renewal, proliferation and differentiation of hematopoietic stem cells, activation of pro-inflammatory and profibrotic mechanisms in clones, as well as increased resistance of clones to external stressors. However, translation of these findings into human medicine requires careful consideration of the fundamental differences in lifespan, metabolism, and immune system organization between model organisms and humans.

Based on the analysis of modern data, we propose an integrative hypothesis according to which inherited genetic variants form an organizational background of predisposition to CDs, while somatic mutations arising and selectively expanding within specific cellular compartments of target organs represent critical events explaining topical specificity, temporal dynamics (age at onset) and variability in the severity of clinical phenotype.

The 9p21 locus containing the tumor suppressor genes CDKN2A and CDKN2B provides a pathogenetic illustration of this relationship. Inherited variants at this locus, associated with coronary heart disease risk, create a state of haploinsufficiency, which manifests in weaker control over cell cycle regulation and DNA repair in vascular smooth muscle cells. However, realization of the full pathogenic potential of this predisposition requires an additional event, such as somatic inactivation of the remaining allele. This initial vulnerability constitutes the essence of a “permissive environment”, since it increases the probability that a somatic event (such as loss of heterozygosity for the remaining wild-type allele) will lead not to cell death, but to its selective advantage and subsequent clonal expansion, finally inactivating the tumor suppressor locus and fixing the proliferative phenotype.

Moreover, in atherosclerotic vessels, activation of the proinflammatory SMC phenotype is associated with epigenome remodeling, in particular, with a decrease in the availability of the ATF3 gene promoter, which is a key transcriptional repressor of inflammation. Notably, germline variants at the ATF3 locus are also associated with coronary artery disease risk according to GWAS (Wang et al., 2022). The combined presence of inherited predisposition and acquired epigenetic dysregulation within the same molecular node highlights the principle of convergence of heterogeneous pathogenic effects on key regulatory elements, which can serve as a general model for explaining the pathogenesis of multiple diseases. This principle of convergence demonstrates that the pathogenesis of CDs is determined not by a simple sum of independent factors, but by their nonlinear dynamic interaction, in which inherited variants create a context that determines the specifics of the response to acquired epigenetic alterations, forming a unique pathological profile for each individual.

Thus, a significant proportion of the population heritability of CDs is explained by the spectrum of frequent inherited genetic variants, and rare, especially somatic mutations make a critical contribution to individual risk by modulating the penetrance of hereditary predisposition and determining the specifics of the pathological phenotype at the level of tissues and organs. Consequently, comprehensive characterization of the continuum of genetic variants, encompassing both inherited polymorphisms and somatic mutations acquired during life, in their dynamic interaction with cell-specific molecular networks in normal and pathological conditions forms a new paradigm of predictive medicine. This paradigm marks the transition from population-based probabilistic risk assessments to causal models of individual pathogenesis based on the principles of clonal somatic evolution.

## Conflict of interest

The authors declare no conflict of interest.

## References

Baylis R.A., Gao H., Wang F., Bell C.F., Luo L., Björkegren J.L.M.,
Leeper N.J. Identifying shared transcriptional risk patterns between
atherosclerosis and cancer. iScience. 2023;26(9):107513. doi
10.1016/j.isci.2023.107513

Benditt E.P., Benditt J.M. Evidence for a monoclonal origin of human
atherosclerotic plaques. Proc Natl Acad Sci USA. 1973;70(6):1753-
1756. doi 10.1073/pnas.70.6.1753

Boyle E.A., Li Y.I., Pritchard J.K. An expanded view of complex traits:
from polygenic to omnigenic. Cell. 2017;169(7):1177-1186. doi
10.1016/j.cell.2017.05.038

Bragina E.Yu., Puzyrev V.P. Genetic outline of the hermeneutics of the
diseases connection phenomenon in human. Vavilovskii Zhurnal
Genetiki i Selektsii = Vavilov J Genet Breed. 2023;27(1):7-17. doi
10.18699/VJGB-23-03

DiRenzo D., Owens G.K., Leeper N.J. “Attack of the Clones”: commonalities
between cancer and atherosclerosis. Circ Res. 2017;
120(4):624-626. doi 10.1161/CIRCRESAHA.116.310091

Dobnikar L., Taylor A.L., Chappell J., Oldach P., Harman J.L., Oerton
E., Dzierzak E., Bennett M.R., Spivakov M., Jørgensen H.F.
Disease-relevant transcriptional signatures identified in individual
smooth muscle cells from healthy mouse vessels. Nat Commun.
2018;9(1):4567. doi 10.1038/s41467-018-06891-x

Fiziev P.P., McRae J., Ulirsch J.C., Dron J.S., Hamp T., Yang Y., Wainschtein
P., … Marques-Bonet T., Rehm H.L., O’Donnell-Luria A.,
Khera A.V., Farh K.K. Rare penetrant mutations confer severe risk of
common diseases. Science. 2023;380(6648):eabo1131. doi 10.1126/
science.abo1131

Happle R. The McCune-Albright syndrome: a lethal gene surviving by
mosaicism. Clin Genet. 1986;29(4):321-324. doi 10.1111/j.1399-
0004.1986.tb01261.x

Happle R. Allelic somatic mutations may explain vascular twin nevi.
Hum Genet. 1991;86(3):321-322. doi 10.1007/BF00202421

Happle R. Mosaicism in human skin. Understanding the patterns and
mechanisms. Arch Dermatol. 1993;129(11):1460-1470

Happle R. Monoallelic expression on autosomes may explain an unusual
heritable form of pigmentary mosaicism: a historical case revisited.
Clin Exp Dermatol. 2009;34(7):834-837. doi 10.1111/j.1365-
2230.2009.03543.x

Jaiswal S., Fontanillas P., Flannick J., Manning A., Grauman P.V.,
Mar B.G., Lindsley R.C., … Atzmon G., Wilson J.G., Neuberg D.,
Altshuler D., Ebert B.L. Age-related clonal hematopoiesis associated
with adverse outcomes. N Engl J Med. 2014;371(26):2488-2498. doi
10.1056/NEJMoa1408617

Kabir I., Zhang X., Dave J.M., Chakraborty R., Qu R., Chandran R.R.,
Ntokou A., … Hwa J., Kluger Y., Martin K.A., Fernández-Hernando
C., Greif D.M. The age of bone marrow dictates the clonality of
smooth muscle-derived cells in atherosclerotic plaques. Nat Aging.
2023;3(1):64-81. doi 10.1038/s43587-022-00342-5

Knudson A.G. Mutation and cancer: statistical study of retinoblastoma.
Proc Natl Acad Sci USA. 1971;68(4):820-823. doi 10.1073/
pnas.68.4.820

Kojima Y., Volkmer J.P., McKenna K., Civelek M., Lusis A.J., Miller
C.L., Direnzo D., … Maegdefessel L., Matic L.P., Hedin U.,
Weissman I.L., Leeper N.J. CD47-blocking antibodies restore phagocytosis
and prevent atherosclerosis. Nature. 2016;536(7614):86-90.
doi 10.1038/nature18935

Koroleva Iu.A., Goncharova I.A., Zarubin A.A., Shipulina S.A., Sleptsov
A.A., Panfilov D.S., Kozlov B.N., Nazarenko M.S. Methylation
levels in the 5′ region of the TBX20 gene in the ascending aorta
change in opposite direction in atherosclerosis and aneurysm. Russ J
Genet. 2024;60(7):977-981. doi 10.1134/S102279542470042X

Limaye N., Wouters V., Uebelhoer M., Tuominen M., Wirkkala R., Mulliken
J.B., Eklund L., Boon L.M., Vikkula M. Somatic mutations in
angiopoietin receptor gene TEK cause solitary and multiple sporadic
venous malformations. Nat Genet. 2009;41(1):118-124. doi 10.1038/
ng.272

Lin A., Brittan M., Baker A.H., Dimmeler S., Fisher E.A., Sluimer J.C.,
Misra A. Clonal expansion in cardiovascular pathology. JACC Basic
Transl Sci. 2023;9(1):120-144. doi 10.1016/j.jacbts.2023.04.008

Luo L., Fu C., Bell C.F., Wang Y., Leeper N.J. Role of vascular smooth
muscle cell clonality in atherosclerosis. Front Cardiovasc Med.
2023;10:1273596. doi 10.3389/fcvm.2023.1273596

Oren O., Libby P. Incorporating management of clonal hematopoiesis
of indeterminate potential into cardiovascular practice. Circulation.
2025;152(14):975-977. doi 10.1161/CIRCULATIONAHA.125.
075769

Pan H., Xue C., Auerbach B.J., Fan J., Bashore A.C., Cui J.,
Yang D.Y., … Califano A., Sims P.A., Zhang H., Li M., Reilly M.P.
Single-cell genomics reveals a novel cell state during smooth muscle
cell phenotypic switching and potential therapeutic targets for
atherosclerosis in mouse and human. Circulation. 2020;142(21):
2060-2075. doi 10.1161/CIRCULATIONAHA.120.048378

Pan H., Ho S.E., Xue C., Cui J., Johanson Q.S., Sachs N., Ross L.S.,
Li F., Solomon R.A., Connolly E.S., Jr., Patel V.I., Maegdefessel L.,
Zhang H., Reilly M.P. Atherosclerosis is a smooth muscle cell-driven
tumor-like disease. Circulation. 2024;149(24):1885-1898.
doi 10.1161/CIRCULATIONAHA.123.067587

Puzyrev V.P., Kucher A.N. Evolutionary ontogenetic aspects of pathogenetics
of chronic human diseases. Russ J Genet. 2011;47(12):
1395-1405. doi 10.1134/S102279541112012X

Schuermans A., Honigberg M.C. Clonal haematopoiesis in cardiovascular
disease: prognostic role and novel therapeutic target. Nat Rev
Cardiol. 2025;22(11):845-856. doi 10.1038/s41569-025-01148-9

Tao W., Yurdagul A., Jr., Kong N., Li W., Wang X., Doran A.C.,
Feng C., Wang J., Islam M.A., Farokhzad O.C., Tabas I., Shi J.
siRNA nanoparticles targeting CaMKIIγ in lesional macrophages
improve atherosclerotic plaque stability in mice. Sci Transl Med.
2020;12(553):eaay1063. doi 10.1126/scitranslmed.aay1063

Wang Y., Gao H., Wang F., Ye Z., Mokry M., Turner A.W., Ye J., …
Björkegren J.L.M., Pasterkamp G., Miller C.L., Ross E.G., Leeper
N.J.
Dynamic changes in chromatin accessibility are associated
with the
atherogenic transitioning of vascular smooth muscle cells. Cardiovasc
Res. 2022;118(13):2792-2804. doi 10.1093/cvr/cvab347

Weldy C.S., Kundu S., Monteiro J., Gu W., Pedroza A.J., Dalal A.R.,
Worssam M.D., … Fischbein M.P., Engreitz J., Kundaje A.B.,
Cheng P.P., Quertermous T. Epigenomic landscape of single vascular
cells reflects developmental origin and disease risk loci. Mol Syst
Biol. 2025;21(11):1522-1546. doi 10.1038/s44320-025-00140-2

